# Recent Advances in the Chemical Synthesis and Evaluation of Anticancer Nucleoside Analogues

**DOI:** 10.3390/molecules25092050

**Published:** 2020-04-28

**Authors:** Mieke Guinan, Caecilie Benckendorff, Mark Smith, Gavin J. Miller

**Affiliations:** 1 Lennard-Jones Laboratory, School of Chemical and Physical Sciences, Keele University, Keele, Staffordshire ST5 5BG, UK; m.guinan@keele.ac.uk (M.G.); c.m.m.benckendorff@keele.ac.uk (C.B.); 2 Medicinal Chemistry Knowledge Center, Stanford ChEM-H, 290 Jane Stanford Way, Stanford, CA 94305, USA; mxsmith@stanford.edu

**Keywords:** nucleoside analogue, anti-cancer, chemical synthesis, heteroatom replacement, chemotherapeutic, prodrug

## Abstract

Nucleoside analogues have proven to be highly successful chemotherapeutic agents in the treatment of a wide variety of cancers. Several such compounds, including gemcitabine and cytarabine, are the go-to option in first-line treatments. However, these materials do have limitations and the development of next generation compounds remains a topic of significant interest and necessity. Herein, we discuss recent advances in the chemical synthesis and biological evaluation of nucleoside analogues as potential anticancer agents. Focus is paid to 4′-heteroatom substitution of the furanose oxygen, 2′-, 3′-, 4′- and 5′-position ring modifications and the development of new prodrug strategies for these materials.

## 1. Introduction 

**Figure 1:**
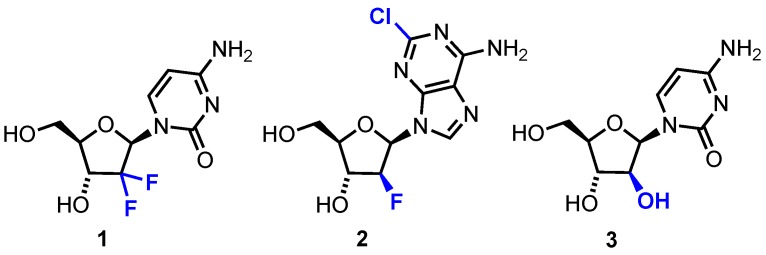
Gemcitabine **1**, clofarabine **2** and Ara-C **3**. Modifications compared to native d-*ribo*-configured purine or pyrimidine nucleosides are shown in blue.

A significant proportion of current chemotherapeutic treatments for cancer involve the use of anti-metabolites, particularly modified nucleoside analogues that possess a capability to mimic native purine or pyrimidine nucleosides which can disrupt metabolic and regulatory pathways [1]. These molecules can be taken up by nucleoside transporters and then phosphorylated to their mono-, di- and triphosphate forms where they are able to interfere with DNA/RNA synthesis and repair; for example, by acting as chain terminators [2] or ribonucleotide reductase inhibitors [3]. Other notable modes of action include epigenetic regulation, through inhibition of DNA regulatory proteins, such as DNA methyltransferase [4]. Selected current examples of anticancer nucleoside analogues approved for chemotherapeutic treatment regimens include capecitabine, gemcitabine **1**, clofarabine **2** and cytarabine (Ara-C) **3** (Figure 1).

Therapeutic intervention using nucleoside analogues is not without its problems and their use is often limited by poor cellular uptake, low conversion to the active triphosphate metabolite, rapid degradation or clearance and development of resistance profiles in certain cell types [5,6]. Consequently, research continues to develop next generations of nucleoside analogues that overcome some of these limitations and provide new therapeutic options. 

This class of antimetabolite also possess a proud history, and current frontline treatment, as antiviral [7,8,9,10,11,12] and, more recently, antibacterial agents [13]. Indeed, the development of nucleoside analogues has a symbiotic relationship between compound class and final therapeutic treatment. For example, gemcitabine **1** was developed as an antiviral, but was subsequently shown to be very toxic to leukaemia cells. 

**Figure 2:**
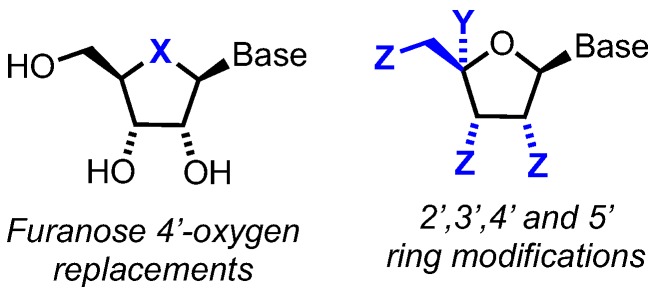
General scope for nucleoside analogues covered in this review. Base = purine or pyrimidine (i.e., C, U, T, A, G or close derivative thereof). X = heteroatom or carbon and Y and Z = ring functional group or modification of native d-*ribo* stereochemistry.

In this review, we survey developments from 2010 onwards for the chemical synthesis and evaluation of modified nucleoside analogues for anticancer research. Specifically, focusing on alterations to the native furanose ring (Figure 2) and generally retaining native purine or pyrimidine nucleobases. Comprehensive reviews concerning hetero-base modifications and general trends in nucleotide synthesis have been covered elsewhere [14,15]. The review is divided into sections that systematically consider: i) furanose 4’-oxygen atom replacements with N, S, Se and C ii) 2′-, 3′-, 4′- or 5′-position furanose ring modifications and iii) new prodrug approaches to deliver nucleoside analogues.

## 2. Furanose Oxygen Atom Replacements

### 2.1. Azanucleosides

Azanucleosides were originally defined as nucleoside analogues where the furanosyl oxygen is replaced by nitrogen, however this group of analogues has been extended to include nucleosides where the resultant pyrrolidine core has been replaced by other nitrogen containing rings, including heterocycles, heterotricycles and acyclic nitrogen-containing nucleosides [16,17]. This class of compound have proven successful in the treatment of cancer [18] and have also been established as having antiviral and antibacterial properties [19]. 

#### Development of Forodesine

**Figure 3:**
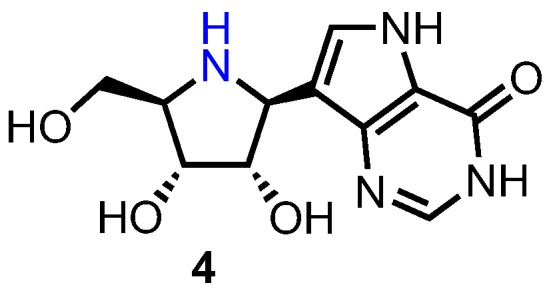
Structure of forodesine **4**.

Purine nucleoside phosphorylases (PNPs) are responsible for the phosphorolytic metabolism of purine nucleosides to ribose/deoxyribose phosphate and the corresponding nucleobase. Patients with abnormally low levels of PNP possess little T-cell immunity due to a severely reduced degradation of deoxyguanosine, which results in the accumulation of the corresponding triphosphate (dGTP). This then reduces the activity of ribonucleotide reductase and induces apoptosis. As such, human PNP inhibitors are potential treatments for T-cell lymphomas [17]. Immucillin H (forodesine) **4** (Figure 3) is a highly potent PNP inhibitor (IC_50_ = 0.48–1.57 nM) which is effective against T-cell malignancies and was found to have excellent oral bioavailability in mice (63%) [20]. 

Forodesine is a gradual onset drug which binds tightly to PNP with a high affinity (K_i_ = 0.023 nM) [21]. Whilst clinical development of **4** was discontinued in the US and Europe, it was recently approved for use in the treatment of relapsed/refractory peripheral T-cell lymphoma (PCTL) in Japan (April 2017) [22]. 

Forodesine is a guanosine analogue and a transition state inhibitor of PNP with 100–1000-fold higher potency than previously identified inhibitors [23]. Due to the combined substitution of the furanosyl oxygen with nitrogen and the *C*-glycosidic bond, **4** is not incorporated into DNA, acting only as a highly selective PNP inhibitor [22]. Also noteworthy is an adenosine mimetic of **4** which is currently under development as a broad-spectrum antiviral [24]. 

**Scheme 1:**
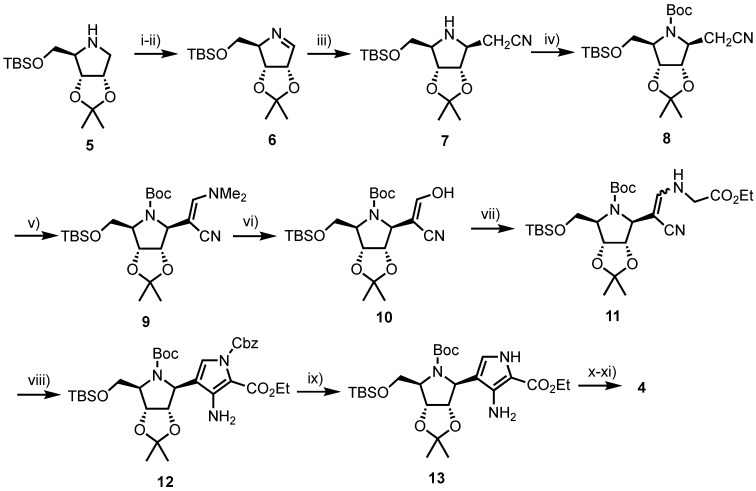
Reagents and conditions: (**i**) NCS, pentane; (**ii**) LiTMP, −78 °C, 36% over two steps; (**iii**) ^n^BuLi, MeCN, THF, −78 °C then tetramethylpiperidine −78 °C, 100%; (**iv**) (Boc)_2_O, CH_2_Cl_2_; (**v**) ^t^BuOCH(NMe_2_)_2_, DMF, 70 °C; (**vi**) THF, AcOH, H_2_O, 72% from **7**; (**vii**) H_2_NCH_2_CO_2_Et^·^HCl, NaOAc, MeOH (**viii**) ClCO_2_Bn, DBU, CH_2_Cl_2_, reflux, 67% from **10**; (**ix**) H_2_, Pd/C, EtOH; (**x**) H_2_NCH=NH^·^AcOH, EtOH, reflux, 91% from **12** and (**xi**) TFA, 81%.

In 2000, Tyler et al. described a linear synthesis of **4** in a satisfactory 39% yield over 10 steps (Scheme 1) [25]. Starting from **5**, synthesised in nine steps by known methods from d-gulonolactone [26], the pyrrolidine was treated with *N*-chlorosuccinimide (NCS), obtaining the 1-chloro anomeric glycoside which subsequently underwent elimination using lithium tetramethylpiperidine (LiTMP) to afford imine **6**. The nucleobase was next assembled via addition of lithiated acetonitrile to afford **7**, followed by protection of the furanosyl nitrogen giving **8** and treatment with Bredereck’s reagent to afford enamine **9**. Acid-catalysed hydrolysis of **9** delivered enol **10** which was reacted with ethyl glycinate to obtain enamine **11**. Treatment of **11** with excess benzyl chloroformate and 1,8-diazabicyclo[5.4.0]undec-7-ene (DBU) revealed **12** and subsequent hydrogenolysis of the *N*-Cbz group provided pyrrole **13**. Completion of the carbocyclic nucleobase was achieved via treatment of **13** with formamidine acetate and acidic removal of the silicon, nitrogen and isopropylidene protecting groups, to afford **4**.

**Scheme 2:**
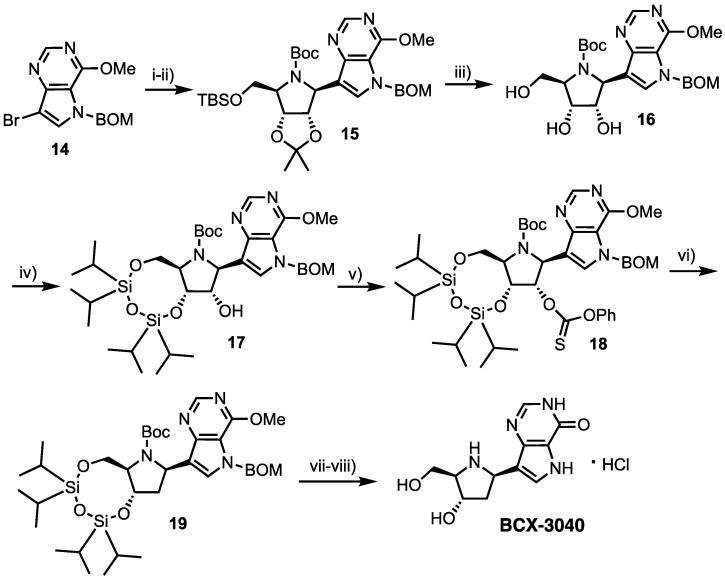
Reagents and conditions: (**i**) **6**, *^n^*BuLi, anisole, ether, −70 °C; (**ii**) (Boc)_2_O, CH_2_Cl_2_, 85% over two steps; (**iii**) 1M HCl, MeOH, 30 °C, 96%; (**iv**) 1,3-Dichloro-1,1-3,3-tetraisopropyldisiloxane, pyridine, 81%; (**v**) *O*-Phenyl chlorothionoformate, MeCN, 90%; (**vi**) 1,1′-Azobis(cyclohexane-1-carbonitrile), toluene reflux, 91%; (**vii**) Pd(OH)_2_, H_2_, EtOH, conc. NH_4_OH, 90% and (**viii**) conc. HCl, MeOH, reflux, 83%.

Forodesine was found to have low oral bioavailability (<11%) in primates, contrary to the case in mice (63% [20]) and was thus originally developed as an intravenous formulation [21]. In 2005, Morris, Jr et al. synthesised **BCX-3040**, the 2′-deoxy analogue of **4**, and comparatively evaluated its oral pharmacokinetic and pharmacodynamic properties in an effort to maintain potency and deliver oral bioavailability [27]. This was hypothesised from 2′-deoxyguanosine exhibiting tight binding to PNP [28] and therefore a possible redundancy for the 2′-OH. Starting from vinyl bromide **14** (Scheme 2), the 9-deazapurine underwent bromine-lithium exchange and addition to imine **6**, obtaining nucleoside **15** in 85% yield. Subsequent removal of 5′-*O*-TBS and 2′,3′-*O*-isopropylidene groups gave **16**, followed by 3’,5’-OTIPDS protection to afford **17**. The free 2′-hydroxyl group was then converted to thiocarbonate **18** and deoxygenated via treatment with 1,1′-azobis(cyclohexane-1-carbonitrile) in excellent yield (91%) to give **19**. Deprotection was completed in two steps, first cleaving the CH_2_OCH_2_Ph (BOM) group, followed by acidic hydrolysis and hydrochloride salt formation to obtain **BCX-3040** in 83% yield (from **19**).

In vitro biological evaluation of **BCX-3040** confirmed it to be a potent PNP inhibitor, with near identical IC_50_ values to **4** (**BCX-3040** IC_50_ = 3.1 ± 0.50 nM and **4** IC_50_ = 1.2 ± 0.21 nM). Administration of 5.0 mg of **4** had a 12.6-fold greater 2′-deoxyguanosine response than administration of 10.0 mg of **BCX-3040**, indicating a reduced bioavailability for **BCX-3040**. Furthermore, following IV administration of 5 mg/kg of **BCX-3040**, the plasma concentration of **BCX-3040** dropped rapidly to 3.0 ± 0.31 µg/mL. Overall, due to the observed rapid clearance and reduced bioavailability of **BCX-3040**, it was concluded to be a poorer PNP inhibitor candidate in comparison to **4**.

### 2.2. Thionucleosides

4′-Thiofuranoses are of known importance in biological systems, for example as chemical biology or biomedical tools [29], and possess chemotherapeutic activity [30,31]. Furthermore, the thioaminal moiety within 4′-thiofuranosyl nucleosides has been proven to be resistant to metabolic hydrolysis in comparison to native 4′-oxa analogues [32]. In the early 1990s, Secrist, Montgomery and co-workers stimulated interest in this class of molecule with their synthesis and biological evaluation of 2′-deoxy-4′-thiopyrimidine nucleosides [30]. Since then, there has been a resurgence of interest in the synthesis and evaluation of these compounds as potential antiviral and chemotherapeutic agents.

#### 2.2.1. 2′-Modfied Thionucleosides

**Scheme 3:**
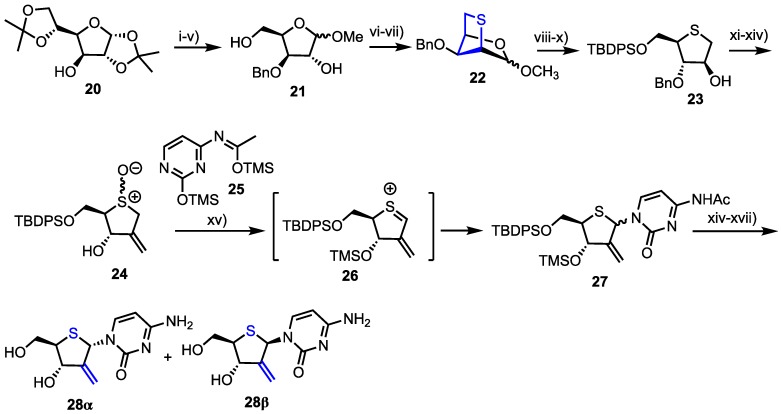
Reagents and conditions: (**i**) BnBr, NaH, DMF, THF; (**ii**) 2M HCl, THF; (**iii**) NaIO_4_, H_2_O, MeOH; (**iv**) NaBH_4_, MeOH, 84% from **20**; (**v**) 5% HCl/MeOH, 91%; (**vi**) MsCl, pyridine; (**vii**) Na_2_S, DMF, α-anomer 78% from **21**, β-anomer 73% from **21**; (**viii**) 4M HCl, THF; (**ix**) NaBH_4_, MeOH, 90% from **2;** (**x**) TBDPSCl, imidazole, DMF, 87%; (**xi**) Ac_2_O, DMSO; (**xii**) Ph_3_PCH_3_Br, NaH, t-amyl alcohol, THF; (**xiii**) BCl_3_, CH_2_Cl_2_, −78 °C then MeOH, pyridine, 92%; (**xiv**) *m*-CPBA, CH_2_Cl_2_, −78 °C, 74% from **23**; (**xv**) **25**, TMSOTf, ClCH_2_CH_2_Cl, 0 °C, 29%; (**xvi**) TBAF, THF and (**xvii**) aqueous NH_3_, MeOH then HPLC separation.

Yoshimura et al. reported the synthesis and biological evaluation of 4’-thia-1-(2-deoxy-2-*C*-methylene-β-d-erythro-pentofuranosyl)cytosine (4’-thio-DMDC) **28β** and 2’-deoxy-2’-fluoro-*arabino*-4’-thiacytidine **33β** as potential antitumour agents [33,34,35]. Their synthesis of **28** started from 1,2,5,6-diisopropylidene-d-glucose **20** and a series of protecting group manipulations delivered 3-*O*-benzyl d-xylose methyl glycoside **21** (Scheme 3). Mesylation of the 2- and 5-hydroxyl groups enabled reaction with sodium sulfide to afford 2,5-bicyclic intermediate **22** as a mixture of anomers. Following conversion of **22** to 4-thio*arabino*furanose **23**, the 2-position hydroxyl group was oxidised and the ketone homologated using a Wittig reagent to install the 2-methylene component, with this material then oxidised to sulfoxide **24**.

The cytidine nucleobase was installed using a Pummerer-type thioglycosylation, via sulphenium ion **26**, which afforded **27** as a mixture of anomers. These were fully deprotected to afford **28α** and **28β** and the desired **28β** isolated using HPLC separation.

**Scheme 4:**
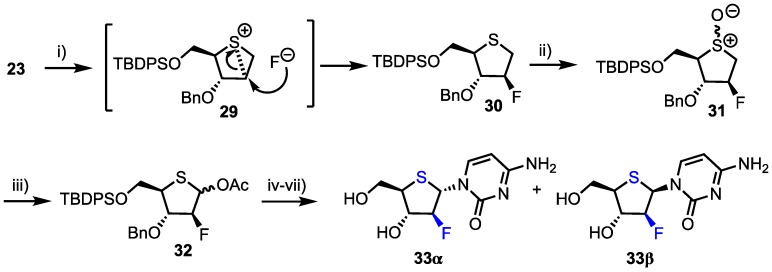
Reagents and conditions: (**i**) DAST, CH_2_Cl_2_, −78 °C, 77%; (**ii**) *m*-CPBA, CH_2_Cl_2_, −78 °C; (**iii**) Ac_2_O, 100 °C, 77% from **23**; (**iv**) **25**, SnCl_4_, MeCN, 93%; (**v**) BBr_3_, MeOH; (**vi**) NH_4_F, MeOH, 60 °C and (**vii**) aqueous NH_3_, MeOH then HPLC separation, 43% (β-anomer) and 17% (α-anomer) from **32**.

Additionally, 2′-deoxy-2′-fluoro-*arabino*-4′-thiacytidine **33** was prepared from intermediate **23** (Scheme 4). Stereospecific DAST fluorination of **23** proceeded through epi-sulphonium intermediate **29** which delivered the 2-deoxy-2-fluoro*arabino* intermediate **30** in 68% yield. *m*-CPBA oxidation to the sulfoxide **31** and subsequent Pummerer rearrangement formed anomeric acetate **32** in 77% yield from **30**. Finally, thioglycosylation was successfully employed to access the corresponding 4’-thionucleoside mixture **33** (54% yield). Comparatively, the group found that using **32** as a donor and employing silyl-Hilbert-Johnson glycosylation conditions yielded **33α/β** in higher yield (93%), but still as a mixture of anomers (2.9:1 α/β). 

**Scheme 5:**
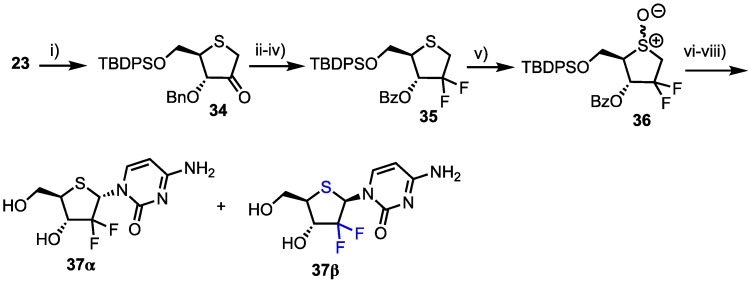
Reagents and conditions: (**i**) Ac_2_O, DMSO; (**ii**) DAST, benzene, 0 °C-r.t., 48%; (**iii**) BCl_3_, CH_2_Cl_2_, −78 °C, then MeOH, pyridine; (**iv**) Bz_2_O, Et_3_N, DMAP, MeCN, 79% from **34**; (**v**) *m*-CPBA, CH_2_Cl_2_, −78 °C; (**vi**) **25**, TMSOTf, ClCH_2_CH_2_Cl, 0 °C, 57% from **35**; (**vii**) TBAF, THF and (**viii**) aqueous NH_3_, MeOH, then HPLC separation, 36% (α-anomer) and 15% (β-anomer) from **36**.

Finally, the group synthesised 2’-deoxy-2’-difluoro-1’(4’-thia-d-ribofuranose)cytosine **37**, a thionucleoside analogue of the potent chemotherapeutic agent gemcitabine **1** (Scheme 5). Intermediate **23** was again utilised and oxidised using Albright-Goldman conditions to obtain ketone **34** which was difluorinated at C2 using DAST. The 3-*O*-benzyl group was next removed and replaced with benzoate to afford **35** in 79% overall yield from **34**. Oxidation to the sulfoxide afforded **36** which subsequently underwent Pummerer rearrangement-glycosylation, before the remaining protecting groups were removed to afford **37α/β** (α/β = 2.4/1) in a moderate yield of 51% from **36**. 

The antineoplastic activities of **28**, **33** and **37** were evaluated and compared to arabinocytidine **3** (Ara-C) and 1-(2-deoxy-2-*C*-methylene-β-d-erythro-pentofuranosyl)cytosine (DMDC) (Table 1). As expected, all the α-anomer forms were found to be inactive against T-cell leukemia CCRF-HSB-2 cells. However, the β-anomers showed considerable cytotoxic activity against the same cell line. Notably, **28β** and **33β** were highly potent against both T-cell leukemia CCRF-HSB-2 cells and human solid tumour KB cells, with IC_50_ values of 0.01 µg/mL (CCRF-HSB-2) and 0.05 µg/mL (CCRF-HSB-2) for **28β** and **33β,** respectively. Indeed, the activity of **28β** was significantly higher than that of its native counterpart, DMDC, against both cell lines, with an IC_50_ value 2.4 times lower in CCRF-HSB-2 cells and 3.7 times lower in KB cells. Interestingly, 4′-thiogemcitabine analogue **37β** had poorer antineoplastic activity compared to **1**, which the authors suggest may be due to a reduction in the phosphorylation efficacy of **37β** by deoxycytidine kinase, a key enzyme which converts 2′-deoxycytidine analogues to their corresponding monophosphates.

#### 2.2.2. 4′-Modfied-2′-deoxythionucleosides

**Figure 4:**
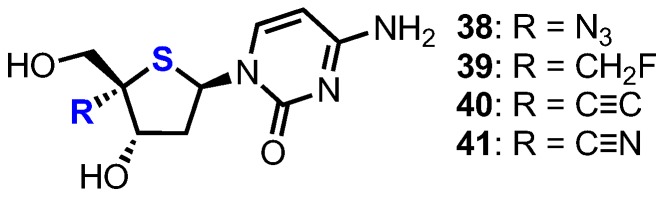
Structures of 2′-deoxy-4’-thiacytidine nucleosides **38**–**41**.

Following earlier work by Parker and colleagues, who identified 4′-thia-2′-deoxycytidine (T-dCyd) as being able to inhibit tumour growth [37], Haraguchi and colleagues reported the synthesis and evaluation of a small library of 4′-position modified 4′-thia-2′-deoxycytidine nucleosides **38**–**41** for their antineoplastic and antiviral activity (Figure 4) [38].

**Scheme 6:**
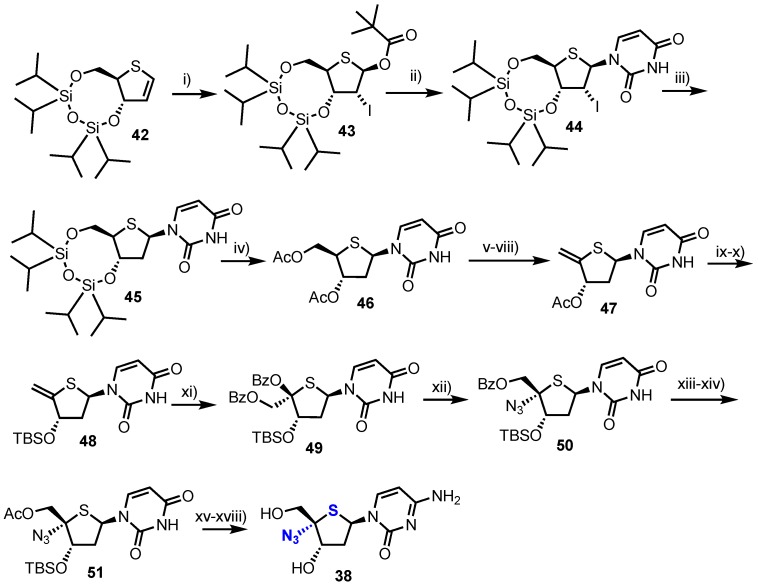
Reagents and conditions: (**i**) NIS, pivalic acid, MeCN/CH_2_Cl_2_, 93%; (**ii**) Uracil, *N*,*O*-bis(trimethylsilyl)acetamide (BSA), TMSOTf, MeCN/CH_2_Cl_2_, 87%; (**iii**) Bu_3_SnH, Et_3_B, toluene, O_2_, −60 °C, 98%; (**iv**) TBAF, Ac_2_O, THF, 100%; (**v**) NH_3_/MeOH; (**vi**) I_2_, PPh_3_, pyridine, dioxane; (**vii**) Ac_2_O, DMAP, DIPEA, CH_2_Cl_2_; (**viii**) BDN, MeCN, 65% over four steps; (**ix**) NH_3_/MeOH; (**x**) TBDMSCl, imidazole, DMF, 61% over two steps; (**xi**) Pb(OBz)_4_, toluene, 63%; (**xii**) TMSN_3_, SnCl_4_, CH_2_Cl_2_, 61%; (**xiii**) NaOMe, MeOH; (**xiv**) Ac_2_O, DMAP, DIPEA, MeCN, 83% over two steps; (**xv**) TPSCl, K_2_O_4_, MeCN, 60 °C; (**xvi**) NH_4_OH, THF; (**xvii**) TBAF, Ac_2_O, THF and (**xviii**) NaOMe, MeOH, 66% over four steps.

Towards analogue **38** the group started from thioglycal **42** (Scheme 6), obtained using established procedures from 2-deoxy-d-ribose in 12 steps [39]. This material was transformed into a glycosyl donor through treatment with *N*-iodosuccinamide (NIS) and pivalic acid, giving one diastereoisomer of 2-iodo derivative **43**. Silylated uracil was then glycosylated with **43** using Vorbrüggen-type conditions to give β-4’-thiouridine **44** which was 2’-deoxygneated via a Bu_3_SnH mediated radical reduction to give **45**. Subsequent TIPDS deprotection, followed by C3’ and C5’-*O*-acetylation delivered **46** to enable a four-step procedure to deliver 4’,5’-unsaturated-4’-thiouridine derivative **47**. *Exo*-thioglycal **47** was next converted to silyl-protected 4’-thionucleoside **48**, which when treated with Pb(OBz)_4_ yielded dibenzoate **49**. S_N_2 inversion back to the native 4’-d-ribo configuration and 4’-azide installation was achieved by reaction with MeSiN_3_ in the presence of SnCl_4_ and afforded the desired 4’-α-azido derivative **50** as the major product. Finally, the nucleoside was converted to the cytidine form via intermediate **51** to give **38** in 7% yield over 18 steps. 

**Scheme 7:**
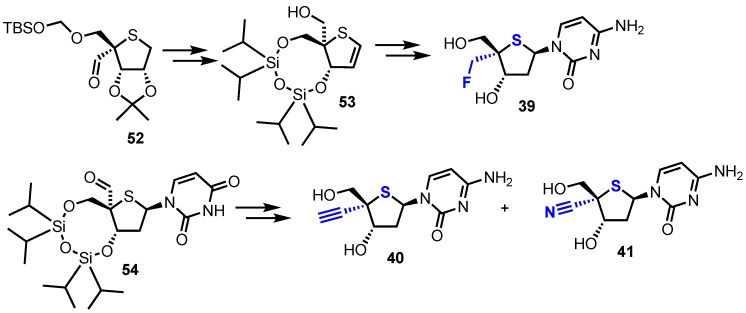
Key intermediates to access 2’-deoxy-4’-thiocytidine nucleosides **39**–**41**.

In order to access 2′-deoxy-4’-*C*-fluoromethyl-4’-thiacytidine **39**, alcohol **53** was prepared from known aldehyde **52** [40,41]. Treatment of **53** with DAST successfully installed the key 4-α-fluoromethyl group which was then elaborated to **39** (Scheme 7). 4-α-alkynyl and nitrile analogues **40** and **41** were synthesised from aldehyde **54** using a late-stage insertion of the key functional group at the 4’-position in the presence of the nucleobase. Of the four 2’-deoxy-4’-modified thionucleosides **38**-**41**, two analogues, **38** and **39**, showed moderate cytotoxicity against human B-cell leukaemia (CCRF-SB; IC_50_ = 7.14 μM and 3.19 μM for **38** and **39** respectively) and human T-cell leukaemia cell lines (Molt-4; IC_50_ = 2.72 μM and 2.24 μM for **38** and **39** respectively). 

### 2.3. Selenonucleosides

**Scheme 8:**
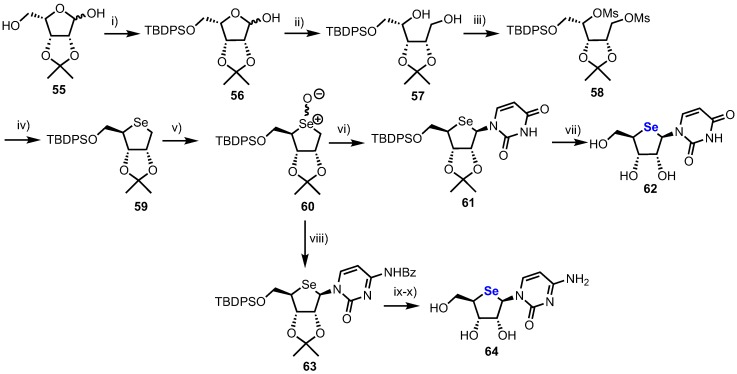
Reagents and conditions: (**i**) TBDPSCl, Et_3_N, DMAP, CH_2_Cl_2_, 92%; (**ii**) NaBH_4_, MeOH, 98%; (**iii**) MsCl, Et_3_N, DMAP, CH_2_Cl_2_, 97%; (**iv**) Se, NaBH_4_, EtOH, THF, 60 °C, 96%; (**v**) *m*-CPBA, CH_2_Cl_2_, –78 °C, 85%; (**vi**) Uracil, Et_3_N, TMSOTf, toluene, CH_2_Cl_2_, 53%; (**vii**) 50% aq. TFA, 81%; (**viii**) *N*^3-^benzoylcytosine, Et_3_N, TMSOTf, toluene, CH_2_Cl_2_, 35%; (**ix**) 50% Aq. TFA and (**x**) NH_3_, MeOH, 82% over two steps.

A first synthesis of pyrimidine 4’-selenonucleosides was reported by Jeong and colleagues in 2008 (Scheme 8), [42] starting from lyxose derivative **55**, synthesised from d-gulonic-γ-lactone in four steps using established procedures [43]. 

Selective protection of the primary alcohol in **55** was achieved by reaction with TBDPSCl, giving hemiacetal **56** which was reduced with NaBH_4_, furnishing diol **57**. Following double mesylation of **57** to give **58**, cyclisation to give 4-selenosugar **59** was achieved by treatment with selenium in the presence of NaBH_4_. Oxidation of **59** to a diastereomeric selenoxide mixture **60** then enabled either a uracil or cytosine nucleobase to be installed via a Pummerer-type glycosylation, furnishing the β-anomers **61** or **63**. 4’-selenouridine **62** was obtained after global deprotection of **61** with 50% aqueous TFA in an overall yield of 12% over 11 steps. 4’-selenocytidine **64** was similarly obtained in an overall yield of 9% over 11 steps. The crystal structure of **62** revealed the non-native ring adopted an unusual C2’-*endo*/C3’-*exo* twist (Southern confirmation), contrary to uridine, which shows a C2’-*exo*/C3’-*endo* twist (Northern conformation). This difference was explained by the introduction of the bulky selenium, whereby stereoelectronic effects observed in 4’-oxanucleosides are outweighed by the size of the heteroatom. 

#### 2’-Substituted-4’-selenoribofuranosyl Pyrimidines

**Figure 5:**
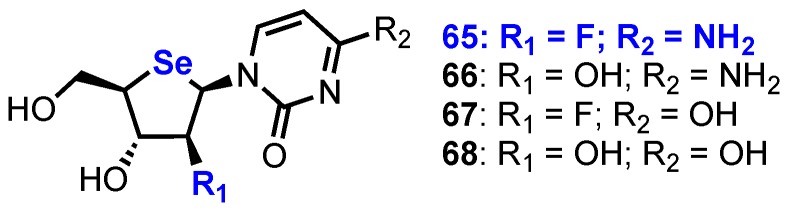
Structures of 2’-substituted 4’-selenoarabinofuranosyl pyrimidine analogues **65**–**68**.

Building on their work in this area, Jeong and co-workers reported the synthesis and biological evaluation of a small library of 2’-substituted 4’-seleno*arabino*furanosyl pyrimidine analogues **65**–**68** (Figure 5) [44,45].

**Scheme 9:**
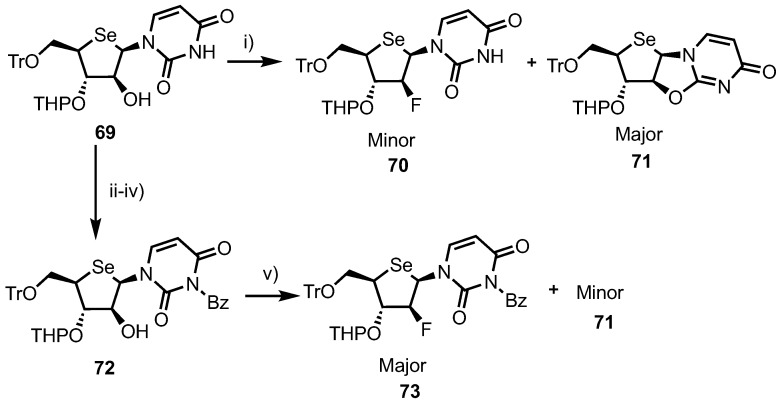
Reagents and conditions: (**i**) DAST, CH_2_Cl_2_, −78 °C, 23%; (**ii**) TESCl, DMAP, CH_2_Cl_2_; (**iii**) BzCl, pyridine, DIPEA, 70 °C; (**iv**) TBAF, THF, 0 °C, 81% over three steps and (**v**) DAST, CH_2_Cl_2_, −78 °C, 45%.

A 2’-fluoroarabino analogue was obtained via a 2’-position DAST fluorination of intermediate **69**, synthesised from 4’-selenouridine **61** using a four-step process [44]. At first attempt, DAST fluorination of **69** led to the formation of the desired 2’-fluoro product **70** as the minor product in 23% yield, along with a major product, 2,2’-*O*-anhydro nucleoside **71**, in 60% yield (Scheme 9). This ratio was subsequently improved by instead fluorinating an *N*^3^-benzoyl derivative **72**, giving **73** in 45% yield, now as the major product, and **71** in reduced 30% yield. 

Following global deprotection and uracil to cytosine conversion, four nucleoside analogues **65**–**68** were evaluated against several human cancer cell lines (HCT116, A549, SU638, T47D, PC-3 and K562) and compared to the established anticancer nucleosides **1** and **3** (Table 2). From this study, it was found that 2’-fluoro-4’-selenoarabinocytidine **65** was the most potent analogue, showing even greater potency than **3**, in the majority of cell lines tested.

**Figure 6:**
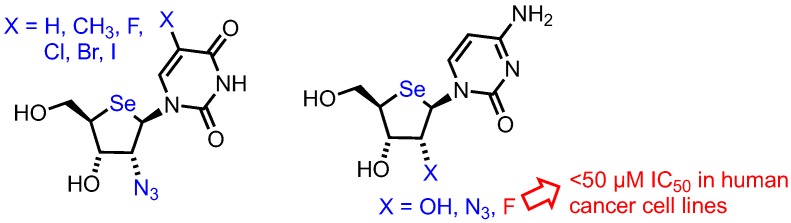
2’-Substituted-4’seleno*ribo*furanosyl nucleosides reported by Jeong.

The group have also reported further 2’-substituted-4’-seleno*ribo*furanosides [46], synthesising a series of pyrimidine 2’-substituted analogues and utilising a 2,2’-*O*-anhydro intermediate to enable regioselective nucleophilic ring opening to the desired *ribo*-configured products (Figure 6). Only a 2’-fluoro *ribo*selenocytosine derivative showed significant activity (uracil, thymine and 5-halo uracil derivatives showed no activity up to 100 µM), but this was less potent than the *arabino*-configured analogue previously described [45]. 

### 2.4. Carbocyclic Nucleosides

**Figure 7:**
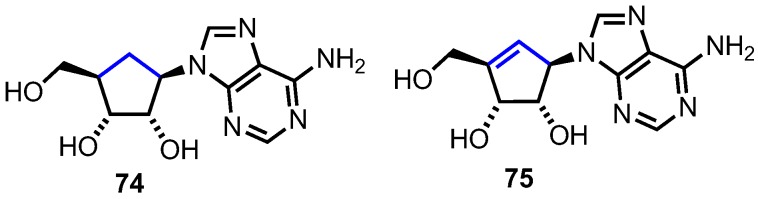
Structures of aristeromycin, **74**, and neplanocin A, **75**.

In carbocyclic nucleosides the furanosyl oxygen is replaced by CH_2_, forming a cyclopentane ring. The lack of a hemiaminal linkage between the nucleobase and the sugar leads to an increased chemical stability. Furthermore, due to the lack of a glycosidic bond, these nucleosides show an enhanced resistance towards phosphorylases [47]. Although considered a second generation of nucleoside analogues, there are two naturally occurring carbocyclic nucleosides, aristeromycin **74** and neplanocin A **75** (Figure 7), both of which exhibit substantial biological activity as antitumor agents [47].

#### 2.4.1. Fluorinated Derivatives of Neplanocin A 

**Figure 8:**
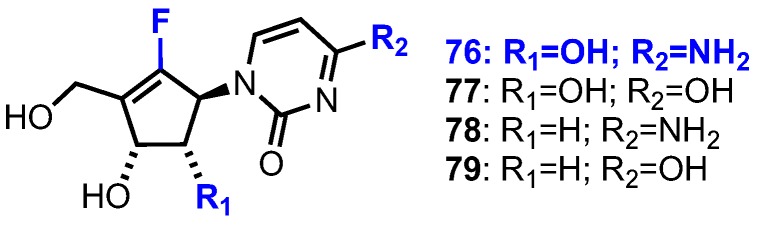
Structures of fluorocyclopentenyl nucleoside analogues **76**–**79**.

In 1988, Driscoll et al. reported that a cytosine analogue (CPE-C) of **75** showed substantial antitumour and antiviral activity [48]. On the basis of these findings, and the continuing clinical success of fluorine containing nucleoside chemotherapeutics [49,50], Jeong et al. subsequently reported the synthesis and biological evaluation of a small library of fluorocyclcopentenyl pyrimidine nucleosides **76**–**79** (Figure 8) [51]. 

**Scheme 10:**
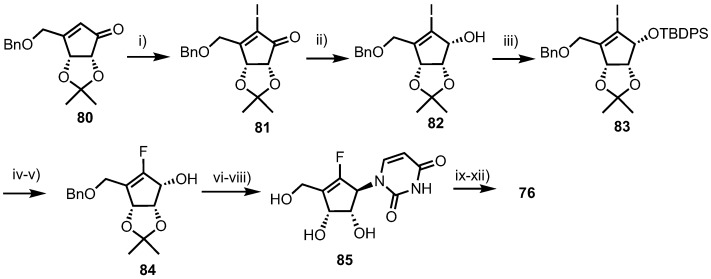
Reagents and conditions: (**i**) I_2_, pyridine, THF, 55%; (**ii**) NaBH_4_, CeCl_3_, MeOH, 93%; (**iii**) TBDPSCl, imidazole, DMF, 97%; (**iv**) NFSI, *^n^*BuLi, THF, −78 °C, 80%; (**v**) TBAF, THF, 80%; (**vi**) *N*^3^-benzoyluracil, DEAD, PPh_3_, THF; (**vii**) NH_3_/MeOH; (**viii**) BBr_3_, DCM, −78 °C, 49% over three steps; (**ix**) Ac_2_O, pyridine; (**x**) POCl_3_, Et_3_N, 1,2,3-triazole; (**xi**) NH_4_OH, 1,4-dioxane and (**xii**) NH_3_/MeOH, 40% over four steps.

Fluorocyclopentenyl cytosine **76** was synthesised from cyclopentenone **80** (Scheme 10), which was obtained from d-ribose in nine steps using established procedures [52,53]. Iodination of **80** was accomplished by treatment with I_2_ and pyridine in THF, to give **81**. Stereo- and regioselective reduction of **76** was achieved using Luche conditions to give **82**, followed by TBDPS protection of the resulting alcohol to deliver **83**. Following lithium-halogen exchange of **83** using *n*-BuLi, electrophilic fluorination was achieved by treatment with *N*-fluorobenzenesulfonimide (NFSI). Anomeric desilylation with TBAF then gave **84** to which a uracil nucleobase was installed via condensation with *N*^3^-benzoyluracil under Mitsunobu conditions. Finally, global deprotection was achieved by sequential treatment with methanolic ammonia and BBr_3_, providing uracil derivate **85** which was converted to **76** via a standard four-step process. 

Of the four nucleoside analogues evaluated (Figure 8), derivative **76** showed significant potency against several human cancer cell lines (Table 3) [51,54,55]. Furthermore, Jeong reported that **76** also showed significant antitumour activity in a nude mouse xenograft model implanted with A549 human lung cancer cells, wherein after 38 days the inhibition of tumour growth was 32% and 58% at 3 and 10 mg/kg doses, respectively [54].

Carbocyclic nucleoside **76** has now been evaluated in more than 100 different cell lines, as well as several xenograft models, showing high potencies against numerous types of cancer, including gemcitabine resistant cell lines [56,57]. Pharmacokinetics and oral bioavailability for **76** were investigated in phase 0 clinical trials, wherein a small cohort of patients were administered a single oral dose (50 mg or 100 mg) of **76**, or a single intravenous dose (20 mg, Table 4) [58]. This study found that the absolute bioavailability for **76** was 56% and 33% for 50 and 100 mg doses, respectively, suggesting it not to be dose-proportional. However, t_1/2_ was found to be 14 h and 21 h for 50 and 100 mg doses, respectively. This may suggest that **76** does exhibit some dose proportionality in some parameters, but not in others and it was noted that this result may be due to the small patient sample size. Analogue **76** is currently in phase II clinical trials for metastatic pancreatic cancer and advanced bladder cancer. 

#### 2.4.2. Norbornane-Derived (C2’,C4’-bridged) Carbocyclic Nucleosides

**Scheme 11:**
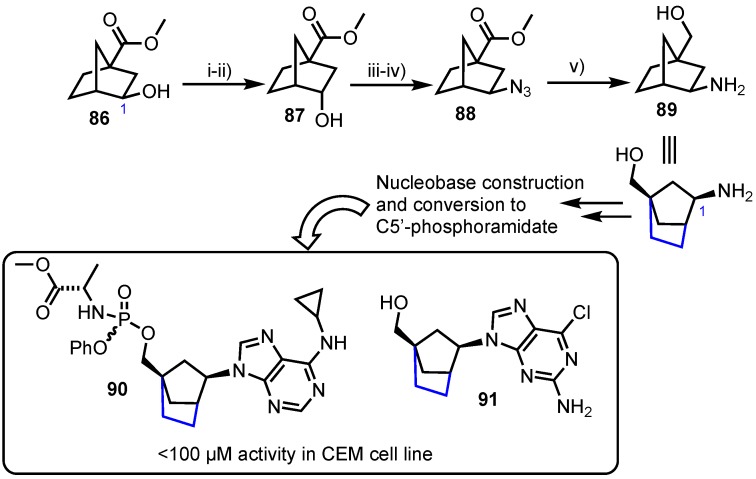
Reagents and conditions: (**i**) PDC, CH_2_Cl_2_, 82%; (**ii**) NaBH_4_, MeOH, 88%; (**iii**) MsCl, pyridine, 99%; (**iv**) NaN_3_, DMF, 115 °C, 92% and (**v**) LiAlH_4_, THF, 59%.

Nencka and colleagues reported the synthesis of norborane, C2’,C4’-bridged carbocyclic nucleosides [59], with a hypothesis of locking the nucleoside analogue in the 2’-exo or North conformation which has garnered attention in the field of carbocyclic antiviral nucleoside analogue development [60]. To access these compounds their synthesis started from known hydroxy ester **86** which was stereochemically inverted at the C1 position via an oxidation-reduction sequence to deliver **87** (Scheme 11). This enabled the desired stereochemistry to be attained at C1 when subsequently inserting an azide by nucleophilic displacement of a mesylate to afford **88** which was reduced to give **89**. With scaffold **89** in hand, the group then elaborated the amine at C1 to several purine and pyrimidine nucleobase forms (including C6 purine analogues) and evaluated their cytotoxic potential. From this series, derivatives **90** and **91** showed IC_50_ activities below 100 *µ*m in human T-lymphocyte (CEM) cells (IC_50_ = 88 *µ*M for **90** and IC_50_ = 78 *µ*M for **91**), but no significant activity was observed in murine leukemia (L1210) or HeLa cell lines. 

#### 2.4.3. C3’,C5’-Bridged Carbocyclic L-Nucleosides

**Scheme 12:**
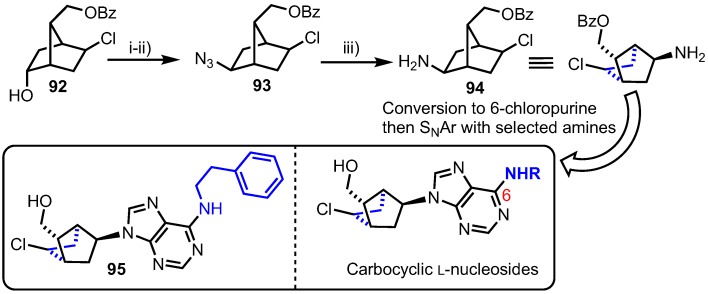
Reagents and conditions: (**i**) MsCl, pyridine, CH_2_Cl_2_, 98%; (**ii**) NaN_3_, DMF, 110 °C, 91% and (**iii**) Pd(OH)_2_/C, MeOH, 87%.

Tănase and colleagues reported the synthesis of an alternative carbocyclic system based on a similar bicyclo[2.2.1]heptane fragment, accessing a small series of 3’-5’-linked L-nucleoside analogues containing C6-amino modifications (Scheme 12) [61]. Their synthesis started from known alcohol **92** and proceeded through azide incorporation (to give **93**) and reduction steps in good yields to deliver amine **94**. The 6-chloropurine ring was then introduced using standard methods and elaborated at C6 with a series of amines via nucleophilic aromatic substitution. This small library was then screened in vitro at a single high dose (10^−5^ M) in the full NCI 58 human tumor cell screen panel. Phenethylamine derivative **95** exhibited growth inhibition of 74% on SK-MEL-5 melanoma and UO-31 renal cancer cell lines, but was not deemed sufficiently cytotoxic for studies to proceed further. The group followed up this report with further synthesis of 6-position carbocyclic analogues [62], derived from **94**, noting that a 6-(4-methoxy-phenethyl)amino group was active, but again the analogue was not progressed further.

## 3. 2’-, 3’- and 5’-Furanose Ring Modifications

### 3.1. 2’-Furanose Modifications

Clofarabine **2** (Figure 1) is a purine nucleoside analogue which, in its corresponding 5’-*O*-triphosphate form, inhibits both ribonucleotide reductase and DNA polymerases and prevents effective DNA synthesis. Ultimately, this leads to cell apoptosis, particularly in rapidly proliferating and dormant cancer cells. The nucleoside analogue exhibits excellent cytotoxic activity in vitro, with an IC_50_ range of 0.028–0.29 *μ*M across a variety of solid tumour and leukaemia cell lines [63], alongside substantial tissue distribution and a half-life of at least 24 h for the active triphosphate metabolite [64]. The use of **2** for treatment of paediatric patients with relapsed or refractory acute lymphoblastic leukaemia was approved by the food and drug administration (FDA) in 2004, the first nucleoside analogue of its kind to be approved in over a decade [65]. 

**Scheme 13:**
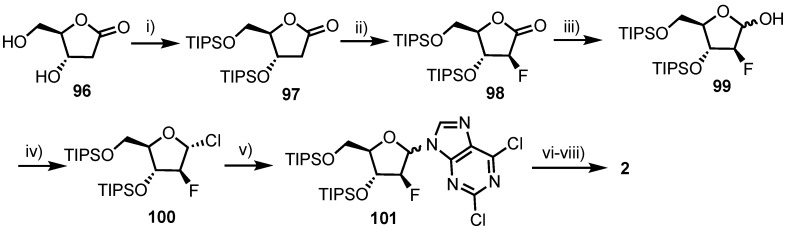
Reagents and conditions: (**i**) TIPSCl, imidazole, DMF, 92%; (**ii**) NFSI, LiHMDS, THF, −78 °C, 72%; (**iii**) DIBAL-H, toluene, −78 °C, 91%; (**iv**) MsCl, Et_3_N, CH_2_Cl_2_, quant.; (**v**) 2,6-dichloropurine, dichloroethane, reflux; (**vi**) NH_3_/isopropanol, sealed tube, 105 °C, 66% over two steps and (**vii**) Me_4_NF, AcOH, DMF, 90%.

In 2010, Sauve and colleagues reported an improved, stereoselective synthesis of **2** [66], in an overall yield of 38% (Scheme 13). This compared favourably to a prior report by ILEX Products Inc., which detailed an overall yield of 14% in six steps, starting from a fully protected ribose derivative. Sauve’s work began from commercially available lactone **96** with 3,5-*O* protection using TIPS affording **97**, which was then diastereoselectively fluorinated at the 2-position, obtaining the *arabino*-configured **98** exclusively in 72% yield. Conversion to the anomeric chloride (via hemi-acetal **99**) delivered **100** which was condensed with 2,6-dichloropurine to afforded **101** in 66% yield over two steps and a β/α ratio of 3.5:1. Access to **2** was gained following diastereomeric separation of **101**, 4-position ammonolysis and deprotection.

### 3.2. 2’-O,4-C’-Bridged Nucleosides

In 2011, Nicolaou and colleagues reported the synthesis and evaluation of a small library of 2’,4’- and 3’,4’-bridged nucleoside analogues, presenting conformationally restrained 3’-endo (North) and 2’-endo (South) systems [67]. These compounds were evaluated for their antiviral, antitumour and antibacterial properties, with 2’,4’-bridged purines **105** and **106** showing µM inhibitory activity against CEM (IC_50_ = 0.36 µM for **105** and 7.6 µM for **106**) and Raji (IC_50_ = 0.25 µM for **105** and 5.8 µM for **106**) cancer cell lines.

**Scheme 14:**
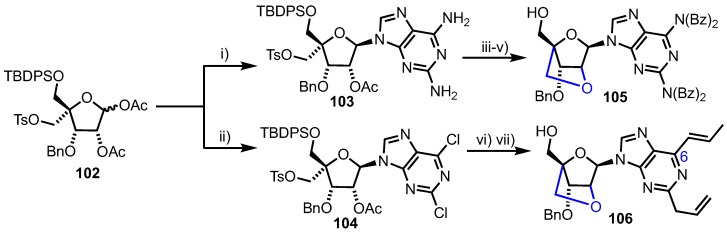
Reagents and conditions: (**i**) 2,6-diaminopurine, BSA, TMSOTf, MeCN, 100%; (**ii**) 2,6-dicholoro-9*H*-purine, BSA, TMSOTf, 64%; (**iii**) NaOH, THF, 94%; (**iv**) BzCl, pyridine, 52%; (**v**) HF, pyridine, 50%; (**vi**) Allyl(tri-*n*-butyl)tin, PdCl_2_(PPh_3_)_2_, DMF then NaOH, THF, 42%, 2 steps and (**vii**) HF, pyridine, 87%.

The synthesis of **105** and **106** derived from a common 4-disubstituted acetate **102**, available using established chemistry from diacetone-d-glucose (Scheme 14) [68,69]. The group used Vorbrüggen glycosylation of either 2,6-diaminopurine or 2,6-dicholoro-9*H*-purine to afford **103** or **104** respectively, with the substitution proceeding in good yields (100% for **103** and 64% for **104**), but noting a requirement to control the amount of *N*,*O*-bis(trimethylsilyl)acetamide (BSA) used to silylate the purine to 2.5 equivalents. 2’,4’-bridged compound **105** was constructed via base mediated cyclisation between C2’ and C4’, followed by per-benzoylation of purine nitrogen and silicon protecting group removal.

To access allyl substituted 2’,4’-system, Stille couplings were completed at positions 2 and 6 of the purine, followed by a similar base-mediated C2’ deacetylation and intramolecular cyclisation to afford, after C5’-protecting group removal, **106**. Isomerisation of the allyl group at C6 of the purine ring was observed during the palladium and base-mediated cyclisation steps. The antitumour activities of **105** and **106** were considerably lower than for the known anticancer nucleoside cladribine (CEM IC_50_ = 0.5 nM and Raji IC_50_ = 9.0 nM), as was the activity (IC_50_ >10 *µ*M) of the 2’4’-bridged analogue of cladribine, suggesting that the inclusion of this conformational restraint or the addition of an extra CH_2_O unit was enough to remove antitumour properties.

### 3.3. 3’-Modified Nucleosides

**Scheme 15:**
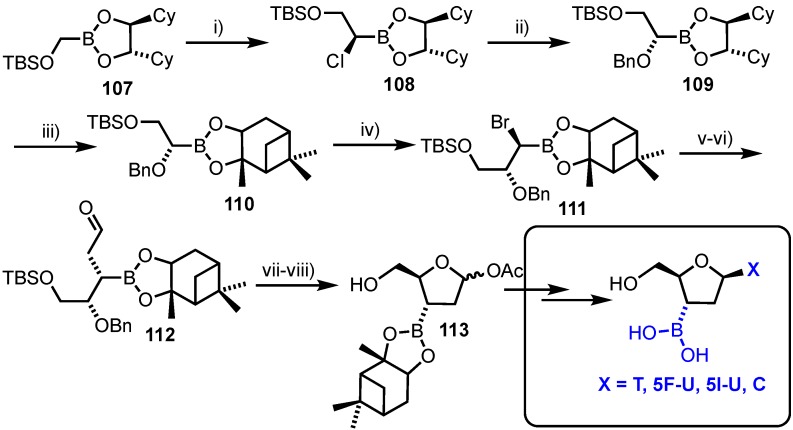
Reagents and conditions: (**i**) LiCHCl_2_, THF then ZnCl_2_; (**ii**) PhCH_2_ONa, 97%; (**iii**) Pinanediol, Et_2_O, H_2_O, 96%; (**iv**) CH_2_Br_2_, LDA, THF then ZnCl_2_, 83%; (**v**) CH_2_CHCH_2_MgBr, 86%; (**vi**) NaIO_4_/K_2_OsO_4_, 2,6-lutidine, dioxane/H_2_O, 63%; (**vii**) H_2_ Pd/C, EtOAc, 81% and (**viii**) Ac_2_O,DMAP, CH_2_Cl_2_, 98%. Cy = cyclohexyl, T = thymine, 5F-U = 5-fluorouracil, C = cytosine, 5I-U = 5-iodouracil.

Cheng and co-workers reported an asymmetric synthesis of 2’,3’-dideoxy-3’-boronic acid pyrimidine nucleosides [70]. The group used two highly diastereoselective reactions of chiral boronic esters with (dihalomethyl)lithium reagents to install the ultimate stereochemistry required at C3’ and C4’ (Scheme 15). Starting from (S,S)-1,2-dicyclohexyl-1,2-ethanediol (DICHED) derived boronate ester **107**, homologation was completed with (dichloromethyl)lithium to afford **108**, the newly formed stereochemistry of which was inverted to give boronate ester **109**. The chiral auxiliary component was next switched from DICHED to a pinanediol (derived from (+)-pinene), delivering **110**. A second diastereoselective homologation was completed with (dibromomethyl)lithium, affording bromo boronic ester **111**. Nucleophilic displacement and inversion of this bromide with allylmagnesium bromide followed by oxidative cleavage of the alkene afforded aldehyde **112**. Hydrogenolysis of the C4-*O*-Bn group in **112**, concomitant cyclisation and anomeric acetylation delivered the final 2-deoxy-*ribo* configured scaffold **113**. This was divergently converted to a series of pyrimidine containing nucleoside analogues containing a unique C3’-boronic acid. Unfortunately, biological evaluation of these compounds demonstrated no significant cytotoxicity towards the HepG2 cell line and all derivatives were observed to undergo gradual hydrolytic cleavage under biological conditions.

More recently, Borbas and colleagues reported a small library of 3’-deoxy-3’-thio substituted *xylo*furanosyl pyrimidines [71]. Utilising a photoinduced thiol-ene reaction the workers were able to effect addition of several different thiols to an appropriately protected 3’-exomethylene ribopyrimidine system. This afforded d-*xylo* configured products in high diastereomeric excess which were shown to have cytostatic activity in the low micromolar range.

### 3.4. C5′-N-Cyclopropylcarboxamido-C6-amino-C2-alkynylated Analogues

**Scheme 16:**
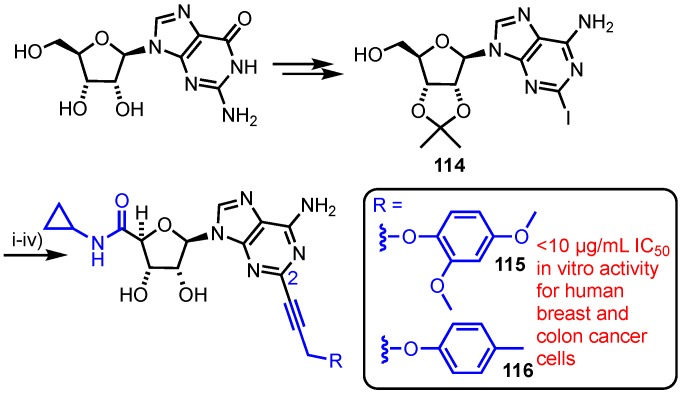
Reagents and conditions: (**i**) KMnO_4_, KOH, H_2_O, 79%; (**ii**) EDC, HOBt, cyclopropylamine, Et_3_N, DMF, 70%; (**iii**) 50% Aq. HCO_2_H, 73% and (**iv**) Pd(PPh_3_)_2_Cl_2_, CuI, Et_3_N, DMF, MeCN, R = **115** = 86%, R = **116** = 85%.

In 2017, Mohan et al. synthesised and evaluated a series of C5′-*N*-cyclopropylcarboxamido-C6-amino-C2-alkynylated purine nucleoside analogues (Scheme 16) [72]. Starting from guanosine, the group accessed C2-aryl iodide derivative **114** which was oxidised at C5’ using KMnO_4_, followed by amide coupling to install the C5′-*N*-cyclopropylcarboxamido group. Following 2’,3’-*O*-acetonide removal, the C2-iodide underwent a series of divergent Sonogoshira couplings to deliver a library of seven purine analogues. From this library, compounds **115** and **116** showed in vitro cytotoxic effects comparable to doxorubicin against human breast (MDA-MB-2312) and human colon (Caco2) cell lines.

### 3.5. 5′-β-Hydroxyphosphonate Analogues

**Figure 9:**
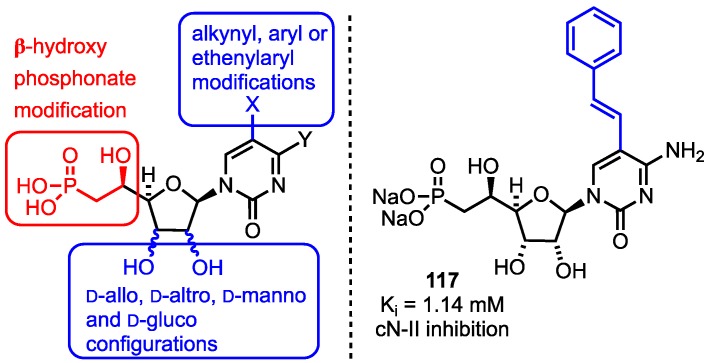
5’-β-Hydroxyphosphonate analogues developed by Peyrottes and active compound **117** from this series, Y = NH_2_ or OH.

In 2014, Peyrottes and co-workers reported their synthesis of a series of β-hydroxyphosphonate nucleosides, targeting 2’ and 3’ hydroxyl group stereochemistry changes and the introduction of 5-position substituents to the nucleobase (Figure 9) [73]. This report built upon the group’s earlier work developing this class of compound as 5’-nucleotidase inhibitors (specifically the cytosolic cN-II 5’-nucleotidase II) [74]. These enzymes catabolise nucleoside 5’-monophosphates and their expression level is of crucial interest for patients undergoing nucleoside analogue chemotherapy, with a higher expression level often associated with a worsened clinical outcome [75].

The group completed the synthesis of a library of 32 β-hydroxyphosphonate analogues which included d-allo, d-altro, d-manno and d-gluco configurations of the furanose 2’- and 3’-positions along with C2’-C3’ ring opened derivatives. They also installed alkynyl, aryl or ethenylaryl groups at C5 of the cytidine or uridine nucleobase using transition-metal cross-couplings of the corresponding C5-iodide. Biological activity data was obtained using recombinant, purified cN-II with inosine monophosphate as substrate. From the resultant SAR study, cytosine-based analogues were generally more active than their uracil counterparts, but the configurational isomers at C2’ or C3’ were generally less active than the parent compounds (allofuranose). Modification of the nucleobase was generally well tolerated with an observed K_i_ value of 1.14 mM for phenylethenyl substituted derivative **117** (Figure 9), noting that high substrate concentrations (in the mM range) are required for activity with cN-II.

### 3.6. Ferronucleosides

**Figure 10:**
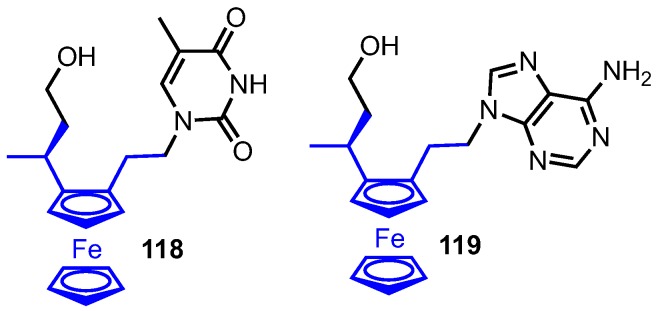
Ferronucleosides developed by Tucker.

Tucker and colleagues recently described their synthesis of ferronucleosides, an important new development in the field of medicinal bioorganometallic chemistry [76]. Here the furanose ring was exchanged for the five-membered cyclopentadienyl ring of a ferrocene unit, but retained the nucleobase and hydroxymethyl group as key components appended in a 2,3 relationship to the ferrocene core (compounds **118** and **119**, Figure 10). Using thymine or adenine as the nucleobase, the compounds were tested with a control series (where the hydroxymethyl or base component were absent) for their cytostatic activity and compared to established chemotherapeutic agents 5-fluorouracil (5F-U) and *cis*platin. In a proliferation activity assay on three tumour cell lines (L1210, HeLa and CEM), both **118** and **119** had activities in the low µM range, with **118** and **119** 20 to 50-fold more active than 5F-U in CEM cell cultures (0.9 µM for **118**, 0.35 µM for **119** versus 18 µM for 5F-U). The compounds also performed promisingly in cell growth inhibition (oesophageal cancer cell line) and cellular viability (MTT assay) studies, with the data indicating that both functional groups appended to the ferrocene component were required for optimum cytostatic activity. 

## 4. Nucleoside Analogue Prodrugs

### 4.1. Phosphorodiamidate Prodrugs

**Scheme 17:**
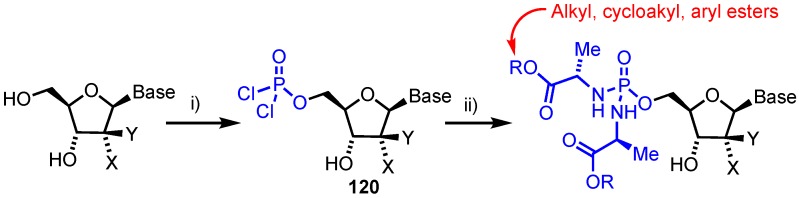
Reagents and conditions: (**i**) (Me)_3_PO_4_, POCl_3_ and (**ii**) Amino acid ester salt, DIPEA. X and Y represent different functional groups for analogue classes e.g. X = Y = F, Base = C = gemcitabine phosphorodiamidate. See Reference [77] for full list of analogues synthesised and evaluated.

In 2018, Slusarczyk’s team reported the synthesis of phosphorodiamidates for a series of established anticancer nucleoside analogues [77]. This was envisioned following the successful ProTide technology developed by McGuigan [78], to deliver nucleoside monophosphate analogues into cells and recent reports of phosphorothioamidate systems [79]. A lack of chirality at phosphorous in a phosphorodiamidate was envisioned to confer advantages in not having to resolve diastereomeric mixtures, often a requirement in earlier generations of ProTides, as S*_P_* and R*_P_* diastereoisomers exhibited markedly different biological activities [80]. Accordingly, seven different anticancer nucleosides (**1**, FUdR, 8-chloroadenosine, fludarabine, AraG, thioinosine and thioguanosine) were converted to their phosphorodiamidate form using a one-pot, two-stage strategy (Scheme 17). First the 5’-OH was converted to a phosphorodichloridate intermediate **120**, followed by double phosphoramidation using an appropriate amino acid ester salt. The panel of analogues were then evaluated in vitro against a wide range of solid tumour and haematological cell lines with the potential for this approach confirmed for FUdR and 8-chloroadenosine, where similar or improved inhibitory activities compared to the parent nucleosides were observed. FUdR and its phosphorodiamidate prodrug showed activity in the sub-micromolar range with IC_50_ values of 0.0046–0.073 µM for FUdR and 0.01–0.40 µM for the FUdR phosphorodiamidates against the wild-type cell lines. Also of note was the inactivity of *arabino* configured analogues, suggesting conversion to the monophosphate might be prevented through their being poor substrates for phosphoramidase activity [81]. Enzymatic studies were undertaken to investigate the bioactivation pathway of this class of nucleoside prodrug with a carboxypeptidase Y assay and ^31^P-NMR confirming the hydrolysis of both esters followed by loss of one phosphoramidate group. 

### 4.2. Vitamin E Phosphate Prodrugs

**Figure 11:**
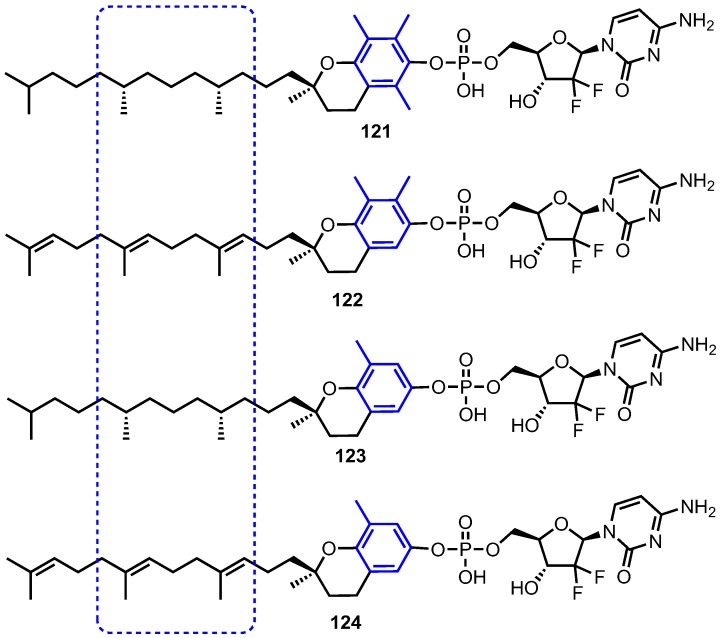
Structures of vitamin E-gemcitabine conjugates **121**–**124**, highlighting changes to the tocopherol or tocotrienol component.

Nucleoside analogue therapy can suffer from inducive and constitutive resistance, which limits the efficacy of the treatment. Isoforms of vitamin E, in particular δ-tocopherol and tocotrienols have displayed anticancer activity. As such, vitamin E phosphate nucleoside analogue prodrugs were envisaged to combat two mechanisms of resistance: i) downregulation of metabolic kinases (deoxycytidine kinase, dCK, in the case of **1**) and ii) nucleoside transport [82,83]. Accordingly, Daifuku and co-workers synthesised and evaluated four isoforms of vitamin E conjugated to **1** (Figure 11, compounds **121**–**124**) [84].

The in vitro GI_50_ of **1**, vitamin E phosphate (VEP) isoforms and compounds **121**–**124** showed these VEP-gemcitabine prodrugs to exhibit anticancer activity, consistent with their catabolism to vitamin E and gemcitabine monophosphate. Conjugate **122** displayed the best activity with GI_50_ values <5 μM in breast MDA (MB-231), non-small cell lung (NCI-H460) and colon (HCT-116) cell lines. The authors suggested this was due to steric hindrance, from the methyl groups proximal to the vitamin E-phosphate bond, reducing the rate of enzymatic cleavage to the monophosphate counterpart.

Prodrugs **122** and **123** were then evaluated in the presence of an inhibitor of nucleoside transport, dipyridamole (DP, Table 5) with the data indicating that both were largely unaffected by the presence of DP in comparison to **1**. This suggested that these prodrugs bypass nucleoside membrane transporters and may thus be beneficial in the treatment of patients with gemcitabine resistant cells. 

In particular, as conjugate **123** displayed significant potency against the three DP(-) cell lines and moderate potency against the DP-dosed cell lines (Table 5), it was selected to further compare activity against **1** in in vitro wild-type leukemic CEM cells and CEM cells deficient in dCK. In dCK(−) cells **1** is not phosphorylated, with GI_50_ values increasing from 0.002 μM in wild-type to 124.5 μM in dCK(*−*). For **123** the GI_50_ increase was significantly lower than that of **1** (from 0.59 μM to 19.2 μM). Furthermore, the half-life of **123** in mice was shown to be 4 h, a 13.9-fold increase to that of **1** (0.3 h [85]). Overall, this study showed an interesting proof of concept for VEP-**1** prodrugs against resistant cancer cell lines. However, further optimisation will be required to obtain promising candidate compounds with increased potencies.

## 5. Conclusions

Nucleoside analogues are an historically accomplished class of drugs with highly diversifiable scaffolds and proven potential to treat a wide range of cancer cell types. Recent synthetic trends have focused on furanose oxygen substitution with heteroatoms or carbon and fluorocyclopentenyl cytosine is proving a promising new clinical candidate in this regard. Alongside this, examples of templating such modifications onto established nucleoside analogue scaffolds, such as Ara-C and forodesine, are emerging. As further pharmacokinetic and pharmacodynamic parameters are evaluated for these architectures, their pharmaceutical utility will be established. Finally, the continued advancement of prodrug strategies to deliver these compounds more effectively and provide options to treat drug-resistant cancer cell types sets an exciting future for nucleoside analogue chemotherapeutics and the underpinning requirement of chemical synthesis in realising this.

## Figures and Tables

**Table 1:** Antineoplastic activities of 2′-modified-4′-thionucleosides.

		Antineoplastic Activities IC_50_ (µg/mL)	
Compound (Anomer)	2′-Substituent	CCRF-HSB-2 ^a^	KB Cells ^b^
**28** **α**	=CH_2_	>10	ND ^c^
**28** **β**	=CH_2_	0.01	0.12
**33** **α**	F (arabino)	>10	ND
**33** **β**	F (arabino)	0.05	0.02
**37** **α**	F_2_	>10	ND
**37** **β**	F_2_	1.5	17
Ara-C **3**		0.05	0.26
DMDC		0.02	0.44

^a^ MTT assay [36]; ^b^ dye uptake method [36]; ^c^ not determined.

**Table 2:** Anticancer activity of 2’-modified 4’-selenoarabino nucleosides **65**–**69** compared to **1** and **3** across several human cancer cell lines.

	IC_50_ (μM)					
Compound	HCT116 ^a^	A549 ^b^	SNU638 ^c^	T47D ^d^	PC-3 ^e^	K562 ^f^
**65**	1.1	0.47	0.14	0.79	0.58	0.63
**66**	7.13	8.83	4.72	ND	ND	86.6
**67**	>100	>100	>100	>100	>100	>100
**68**	>100	>100	>100	>100	>100	>100
**3**	5.30	1.90	0.15	2.70	55.9	0.05
**1**	0.01	0.09	ND	ND	0.04	ND

Human cancer cell tissue type ^a^ colon; ^b^ lung; ^c^ stomach; ^d^ breast; ^e^ prostate; ^f^ myelogenous leukemia.

**Table 3:** Anticancer activity of **76** in human cancer cell lines.

	Cancer Cell Line						
	HTC-116 ^a^	MDA-MB-231 ^b^	PANC-1 ^c^	MCF-7 ^d^	A549 ^e^	MKN45 ^f^	U251 ^g^
**IC_50_ (μM)**	0.39	0.18	0.62	0.34	0.34	0.50	0.83

Human cancer cell tissue type ^a^ colon; ^b^ breast; ^c^ pancreatic; ^d^ hormone-dependent breast; ^e^ lung; ^f^ stomach; ^g^ brain.

**Table 4:** Pharmacokinetic data for **76** from phase 0 clinical studies.

Dose (mg)	Tmax (h)	Cmax (ng/mL)	t_1/2_ (h)	Oral Bioavailability (%)
20 *	0.3	1144	-	-
**50**	2.2	303	14	56
**100**	2.5	311	21	33

* Intravenous.

**Table 5:** GI_50_ values of **1**, **122** and **123** in the presence or absence of dipyridamole.

			Cancer Cell Line			
	Breast MDA-MB-231 (μM)		Non-Small Cell Lung NCI-H460 (μM)			Colon HCT-116 (μM)
**Compound**	DP (−)	DP (20 *μ*M)	DP (−)	DP (20 *μ*M)	DP (−)	DP (20 μM)
**1**	3.08	56.8	0.02	0.82	0.03	2.39
**122**	30.3	27.8	7.16	16.0	5.55	12.6
**123**	17.2	23.3	2.14	1.47	3.07	6.74
